# Self-assembled GLP-1/glucagon peptide nanofibrils prolong inhibition of food intake

**DOI:** 10.3389/fendo.2023.1217021

**Published:** 2023-07-24

**Authors:** Myriam M. Ouberai, Ana L. Gomes Dos Santos, Sonja Kinna, David C. Hornigold, David Baker, Jacqueline Naylor, Lihuan Liang, Dominic J. Corkill, Mark E. Welland

**Affiliations:** ^1^ Nanoscience Centre, Department of Engineering, University of Cambridge, Cambridge, United Kingdom; ^2^ Advanced Drug Delivery, Pharmaceutical Sciences, BioPharmaceuticals R&D, AstraZeneca, Cambridge, United Kingdom; ^3^ Cardiovascular, Renal and Metabolic Diseases, BioPharmaceuticals R&D, AstraZeneca, Cambridge, United Kingdom; ^4^ Cardiovascular, Renal and Metabolic Diseases, BioPharmaceuticals R&D, AstraZeneca, Gothenburg, Sweden; ^5^ Bioscience In Vivo, Research and Early Development, Respiratory & Immunology, BioPharmaceuticals, R&D, AstraZeneca, Cambridge, United Kingdom

**Keywords:** nanofibrils, GLP-1/glucagon, peptides, depot formulations, self-assembly, metabolic diseases

## Abstract

**Introduction:**

Oxyntomodulin (Oxm) hormone peptide has a number of beneficial effects on nutrition and metabolism including increased energy expenditure and reduced body weight gain. Despite its many advantages as a potential therapeutic agent, Oxm is subjected to rapid renal clearance and protease degradation limiting its clinical application. Previously, we have shown that subcutaneous administration of a fibrillar Oxm formulation can significantly prolong its bioactivity *in vivo* from a few hours to a few days.

**Methods:**

We used a protease resistant analogue of Oxm, Aib2-Oxm, to form nanfibrils depot and improve serum stability of released peptide. The nanofibrils and monomeric peptide in solution were characterized by spectroscopic, microscopic techniques, potency assay, QCM-D and *in vivo* studies.

**Results:**

We show that in comparison to Oxm, Aib2-Oxm fibrils display a slower elongation rate requiring higher ionic strength solutions, and a higher propensity to dissociate. Upon subcutaneous administration of fibrillar Aib2-Oxm in rodents, a 5-fold increase in bioactivity relative to fibrillar Oxm and a significantly longer bioactivity than free Aib2-Oxm were characterized. Importantly, a decrease in food intake was observed up to 72-hour post-administration, which was not seen for free Aib2-Oxm.

**Conclusion:**

Our findings provides compelling evidence for the development of long-lasting peptide fibrillar formulations that yield extended plasma exposure and enhanced *in vivo* pharmacological response.

## Introduction

Obesity is a complex multifactorial chronic disorder estimated to affect 20% of the world’s adult population by 2030 (1). It is characterized by the accumulation of excess body fat and can develop into a treatment-refractory condition that affects physical function, mental health and quality of life. Likewise, the number of people with type 2 diabetes (T2DM) has more than doubled over the past three decades and it has been estimated that over 400 million people could be affected by 2030 (2). To address this, pharmaceutical companies are focusing efforts on the development of new treatments with improved efficacy and safety as well as critically enhancing patient compliance and comfort. Peptides derived from gut hormones have been reported to reduce gut motility, decrease appetite and facilitate nutrient disposal (3), and synthetic derivatives of these, including dual and triple pharmacology variants, are currently being widely developed to treat obesity and T2DM (4). For example, glucagon and glucagon-like peptide-1 (GLP-1) receptor agonists are being explored as therapeutic agents to reduce both blood glucose levels and body weight (3, 5). One naturally occurring dual agonist peptide is oxyntomodulin (Oxm), a member of the glucagon family which has been shown to suppress food intake and increase energy expenditure in both rodents and patients (6–8). The poor bioavailability profile of Oxm, with an elimination half-life of 12 min in humans, hinders its use as therapeutic agent as Oxm would need to be administered as a continuous infusion to maintain its effect. To address this limitation, we have explored the natural propensity of Oxm to self-assemble reversibly into nanofibrils. Oxm nanofibrils act as a slow release depot after subcutaneous (s.c.) administration to significantly prolong peptide presence in serum from a few hours to days, as compared to the administration of free peptide (9). When tested in mice, the nanofibril formulation significantly lowered blood glucose levels for up to 6 h after administration and showed an improvement in glucose control (9). However, upon release of free peptide from the fibrils, Oxm is still subject to rapid proteolytic degradation by proteases such as DPP-4 limiting the pharmacologically active peptide concentration in serum (10). Here, we extend our work by incorporating a discrete change to the amino-acid sequence of Oxm to confer resistance against protease degradation and increase the serum stability of free peptide upon release from the fibrils. The serine residue in position 2 is replaced by the aminoisobutyric acid (Aib) residue which, although not one of the 20 amino acid residues found in mammalian proteins, is a common residue occurring in microbial peptides. Aib is considered as a strong helix inducer residue as evidenced by its ability to promote helical secondary structure in synthetic and natural sequences (11). The variant Aib2-Oxm has been shown to be resistant to DPP-4 degradation and stimulate cAMP production (12). *In vivo*, Aib2-Oxm significantly increased overall plasma insulin levels and induced significant appetite suppressive effects in mice (12). In this study, we characterize the self-assembly propensity of Aib2-Oxm in comparison to Oxm and assess the effect of s.c. administration of Aib2-Oxm fibrils in rodents on peptide exposure and food intake.

## Results

### Aib2-Oxm fibril formation and structural characterization

The first stage of Aib2-Oxm self-assembly was carried out as previously described for Oxm, at a concentration of 10 mg mL^-1^ with the pH adjusted to 7 in 0.09% saline (9). The self-assembly process was stopped when the solution turned turbid, after 3 to 6 days of agitation under orbital shaking at 37°C and 2 to 3 days under quiescent conditions. Similar to Oxm, the conversion yield was estimated to be 99% under these conditions (9). The fibrils were then used as seed fibrils in order to promote fibril elongation and the formation of a gel-like suspension. First, 1% (by mass) of the fibrillar material was used to seed a freshly prepared 10 mg mL^-1^ Aib2-Oxm peptide solution in water. However, after 7 days incubation at 37°C under quiescent conditions, only 12% conversion yield was measured whereas approximately 28% can be achieved with Oxm peptide under similar conditions (9). The dilution of the fibril solution to 1 mg mL^-1^ in 0.09% saline failed to achieve a conversion yield >90% as observed for Oxm and only 30% conversion yield was measured after 9 days at 37°C.

To improve the conversion yield, saline and seed fibril contents were increased, and the solutions were incubated at RT. After 12 days incubation of an Aib2-Oxm solution at 10 mg mL^-1^ in 0.09% saline with 10% (by mass) seed fibrils, more than 80% conversion yield was achieved. Then the fibril solution was diluted to concentrations of 1 and 4 mg mL^-1^ in saline at concentrations ranging from 0.09 to 0.36% and incubated at RT to achieve conversion yields higher than 90% and a turbid gel-like suspension. These results indicate that the elongation process for Aib2-Oxm peptide is less favourable than Oxm peptide, as more seed fibrils and higher ionic strength solutions are required to achieve a high conversion yield.

**Figure 1:**
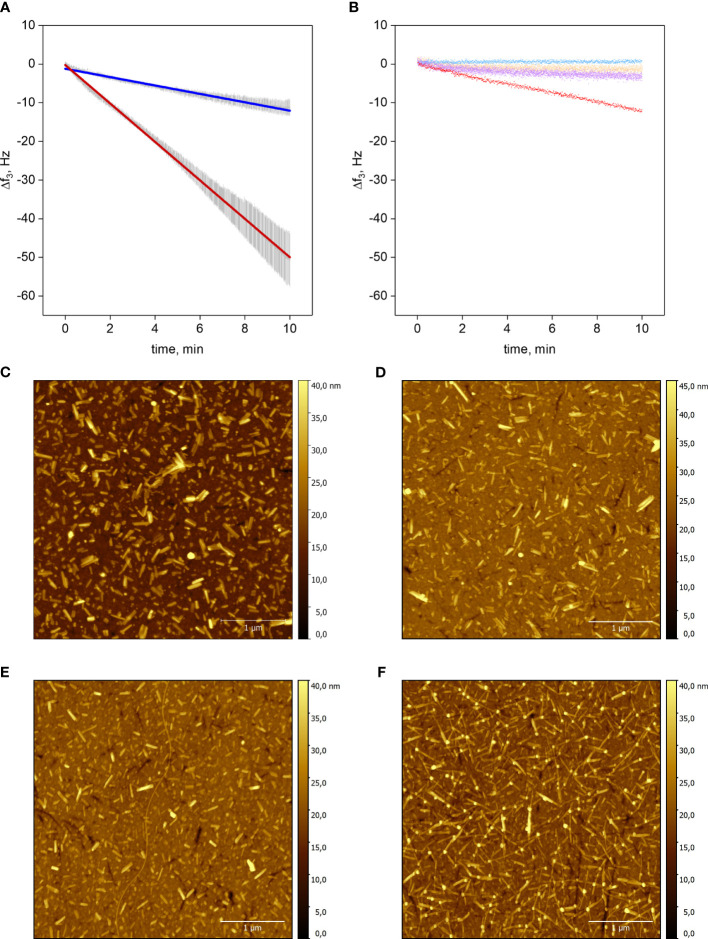
Elongation of Aib2-Oxm and Oxm fibrils. a-b, Resonance frequency shift f3 as a function of time of the silicon dioxide QCM chip upon incubation of peptide. Elongation of Aib2-Oxm fibrils (dark blue) or Oxm (red) fibrils with 2 mg mL-1 Aib2-Oxm or Oxm, respectively **(A)**. Linear approximation ± SD (n=3), R2 = 0.99. Control experiments: Aib2-Oxm fibrils incubated with 0.09% NaCl (blue); empty SiO2 chip incubated with 0.5 mg mL-1 Oxm (orange); Aib2-Oxm fibrils incubated with 0.5 mg mL-1 Oxm (violet); Oxm fibrils incubated with 0.5 mg mL-1 Oxm (red) **(B)**. c-f, Representative AFM images of QCM-D sensors. Oxm fibrils before **(C)** and after incubation **(D)** with 2 mg mL-1 Oxm in 0.09% saline, and Aib2-Oxm fibrils before **(E)** and after **(F)** incubation with 2 mg mL-1 of Aib2-Oxm in 0.09% saline. Scale bar, 1 μm.

We performed QCM-D studies to quantify the difference in the elongation rate between Oxm and Aib2-Oxm (**Figure 1**). QCM-D is a *quasi*-real-time, highly sensitive, label-free biosensing technique that measures the resonance frequency of a quartz sensor. Increase of the resonator`s mass, e.g. by adsorption or binding of molecules, results in damping of the resonance frequency. Elongation of fibrils on the chip surface results in linear frequency decrease, where the slope is a measure for the elongation rate (13).

Short fibrils of Oxm and Aib2-Oxm were obtained by sonication of mature fibrils and were physically adsorbed onto silicon dioxide QCM-D sensors. The elongation process was monitored by a decrease in the resonance frequency during incubation with freshly prepared peptide solutions (**Figures 1A, B**). Incubation of empty chips with 0.5 mg mL^-1^ Oxm solution caused a very shallow decrease in frequency shift (~0.11 Hz min^-1^), showing that formation of multilayers or aggregation on the surface is negligible. Conversely, when 0.5 mg mL^-1^ Oxm solution was passed over Oxm seed fibrils resulted in an approximately linear frequency decrease at a slope of -1.18 Hz min^-1^. Interestingly, a comparable frequency shift slope of -1.09 Hz min^-1^ was found for Aib2-Oxm elongation at an Aib2-Oxm solution concentration as high as 2 mg mL^-1^. Oxm fibril elongation at 2 mg mL^-1^ caused a frequency shift slope of -4.97 Hz min^-1^, indicating an elongation rate 4.5 times faster than Aib2-Oxm fibril elongation under the same conditions. The variation in mass on the top of the chip was confirmed to be an elongation process as observed by AFM, where longer fibrils were observed for Oxm and Aib2-Oxm fibrils following incubation with their respective peptides (**Figures 1C–F**).

Aib2-Oxm fibrils incubated in 0.09% saline did not result in loss of mass in an analogous setup, demonstrating that slower association, not faster dissociation, is responsible for the overall decreased elongation rate of Aib2-Oxm fibrils. We also tested if Aib2-Oxm fibrils can be elongated by fresh Oxm. The slope was about 4-fold less steep than for comparable Oxm fibril elongation, and not significantly steeper than the adsorption slope on an empty sensor. We conclude that Aib2-Oxm fibrils only weakly cross-seed Oxm.

**Figure 2:**
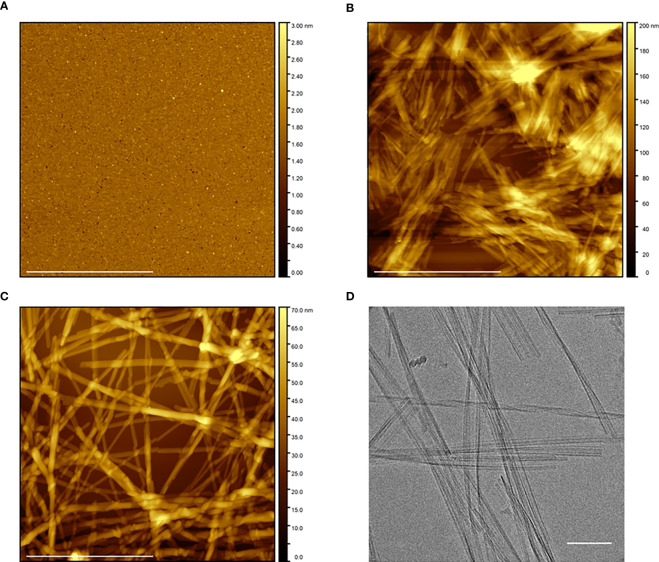
Morphological characterization of free and fibrillar Aib2-Oxm. a–c, AFM images of Aib2-Oxm peptide freshly dissolved at 10 mg mL-1 in 0.09% saline **(A)**, after 5 to 6 days of incubation with orbital shaking and 2 to 3 days under quiescent conditions **(B)**, and after incubation of 1 mg mL-1 fibrils with a 10 mg mL-1 free peptide solution in 0.09% saline for 12 days and diluted at 1 mg mL-1 in 0.09% saline. Scale bar, 1 μm **(C)**. **(D)** CryoEM of fibrils at 1 mg mL-1 in 0.09% saline prepared in similar conditions as described in **(C)** Scale bar, 100 nm.

We next characterized the overall morphologies of Aib2-Oxm species by AFM and CryoEM imaging techniques. AFM analysis of fresh Aib2-Oxm showed a homogeneous and smooth layer of peptide that was deposited onto mica (**Figure 2A**). As observed for Oxm, upon agitation of Aib2-Oxm peptide under orbital shaking, the AFM analysis showed the presence of short fibrils (< 1 μm) grouped into bundles (**Figure 2B**). Following the elongation process at 10 mg mL^-1^ with 10% (by mass) seed fibrils in 0.09%, the species appeared as long fibrillar structures of at least 2 µm in length and approx. 8 nm in height for single fibrils (**Figure 2C**). Interestingly, further characterization of these fibrils by CryoEM showed the presence of twisted fibrils and ribbon-like structures (**Figure 2D**).

**Figure 3:**
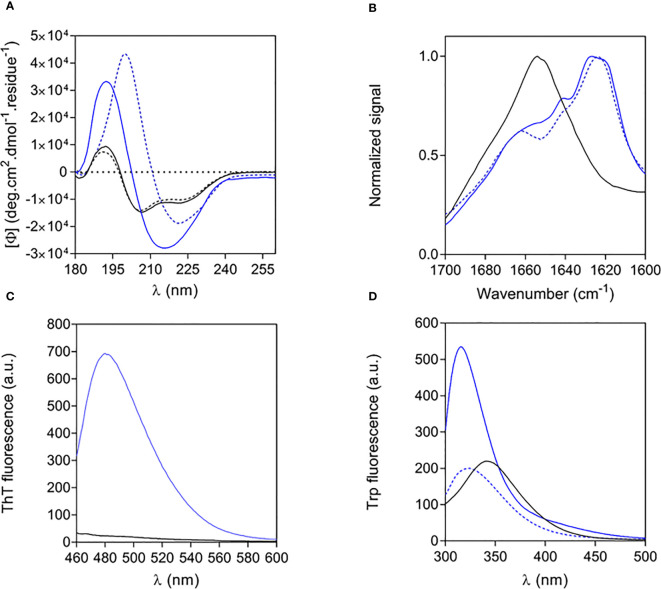
Structural properties of free and fibrillar Aib2-Oxm compared to Oxm species **(A, B)** and of free to fibrillar Aib2-Oxm **(C, D)**, Free (black) and fibrillar (blue) Aib2-Oxm (line) and Oxm (dashed line) at 1 and 2 mg mL-1 in 0.09% saline. Far-UV CD **(A)** and ATR FT-IR **(B)** spectra, ThT **(C)** and Trp **(D)** fluorescence spectra.

The structural properties of free and fibrillar Aib2-Oxm peptide were characterized by a set of complementary biophysical techniques and compared to Oxm structural properties. We first assessed the secondary structure content of free and fibrillar Aib2-Oxm peptides. CD spectra showed that free Aib2-Oxm peptides were mainly arranged in an α-helical conformation (40% α-helical and 10% β-sheet content), in a similar content than the one observed with Oxm (**Figure 3A**). However, compared to Oxm, Aib2-Oxm showed a less marked change in conformation towards β-sheet structure when forming fibrils as shown by the CD spectra (**Figure 3A**). The conformational change of Aib2-Oxm peptide was further assessed by attenuated total reflection (ATR) Fourier transform infrared (FT-IR) spectroscopy (**Figure 3B**). Similar to Oxm, the analysis of the ATR-FT-IR data suggests that free Aib2-Oxm is composed of disordered and α-helix structures as indicated by the band at 1650-1655 cm^-1^. By contrast, fibrillar Aib2-Oxm displayed a maximum at 1624 cm^-1^ characterizing β-sheet content but the spectrum also showed prominent shoulders at 1655 and 1640 cm^-1^ which indicates that Aib2-Oxm peptides adopt a different conformational state than Oxm when forming the fibrils with a significant α-helical content. When the probe Thioflavin T (ThT) was applied to fibrillar Aib2-Oxm, a characteristic fluorescence emission at 480 nm was observed (**Figure 3C**). Tryptophan fluorescence was then monitored in order to probe the environment surrounding the tryptophan residue in the free and fibrillar Aib2-Oxm peptides. As expected, fibrillar Aib2-Oxm showed a blue shift in tryptophan fluorescence with a λmax at 314 nm compared to free Aib2-Oxm (λmax = 343 nm) (**Figure 3D**), which suggests that fibrillar peptide is surrounded by a much more hydrophobic environment than free peptide. Interestingly, this blue shift is more intense than free Aib2-Oxm and more marked than the one observed for fibrillar Oxm (λmax = 324 nm).

Altogether, these structural data show that Aib2-Oxm is adopting a different conformation and packing in the fibrils compared to Oxm peptide. A full elucidation of the molecular details underlying these structural differences is current under investigation.

### Aib2-Oxm nanofibrils display a higher propensity to dissociate than Oxm nanofibrils

**Figure 4:**
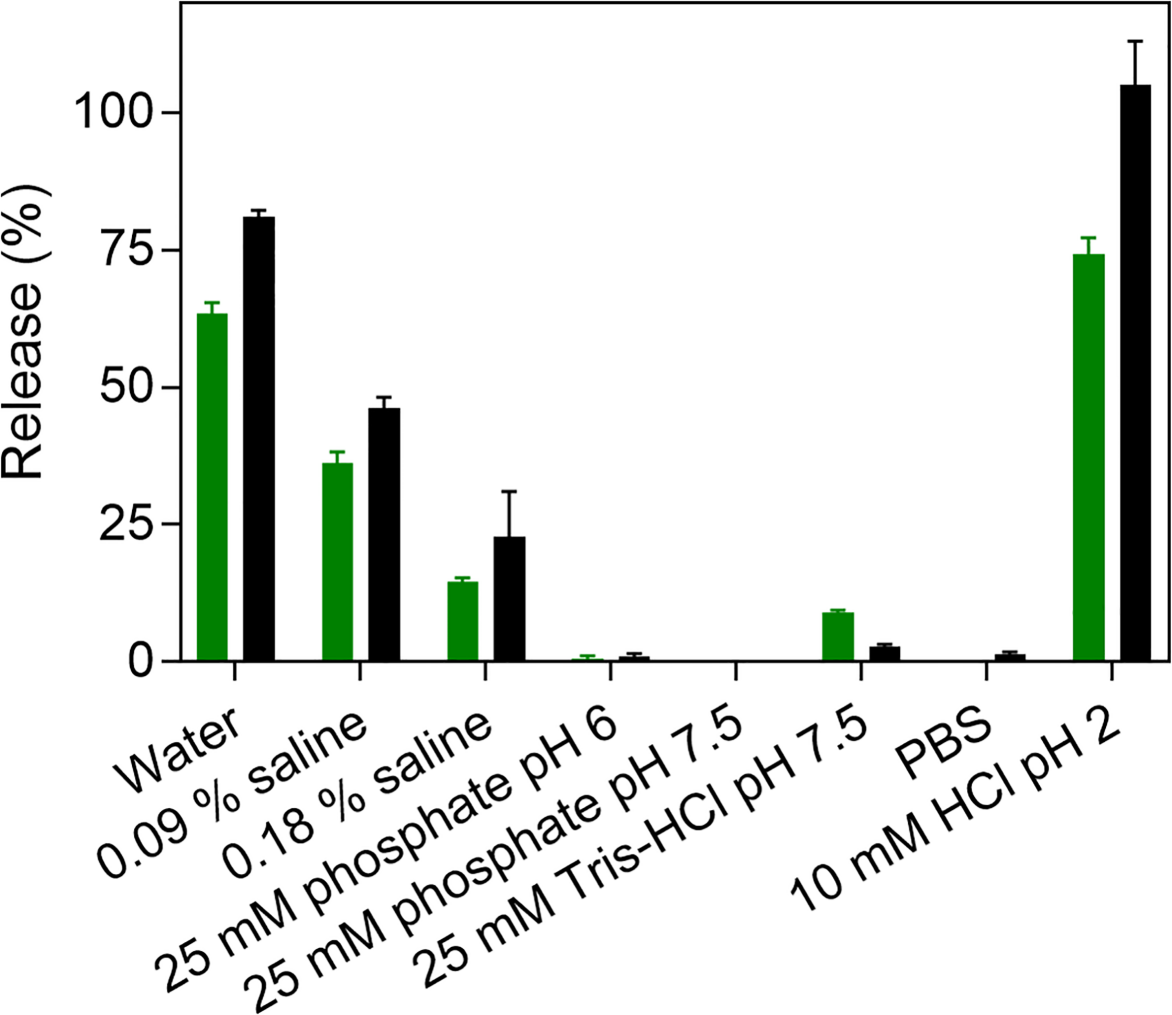
Dissociation of fibrillar Aib2-Oxm. Peptide release (%) after incubation of 1 mg mL-1 fibrillar Aib2-Oxm in eight media and incubated for 4 h (green) and 48 h (black) at 37°C.

The dissociation of fibrillar Aib2-Oxm at 1 mg mL^-1^ was assessed after incubation for 4 and 48 h in eight different conditions: water, 0.09% saline, 0.18% saline, phosphate buffer (25 mM, pH 6), phosphate buffer (25 mM, pH 7.5), Tris-HCl buffer (25 mM, pH 7.5), phosphate-buffered saline (PBS; pH 7.4) and aqueous HCl (10 mM, pH 2) (**Figure 4**). After 4 h, more than 50% of the peptide was released from the fibrils in water and in HCl. Interestingly, while fibrillar Oxm was stable in 0.09% saline, more than 30% of released Aib2-Oxm was measured after 4h and 48h incubation (9). Increasing saline content from 0.09% to 0.18% appears to stabilize the fibrillar state with 23% of released peptide detected after 48h when 46% was measured in 0.09% saline.

The effect of pH was investigated by incubating the fibrils in phosphate buffers (25 mM) at pH 6 and pH 7.5 (**Figure 4**). In both conditions, only traces of released peptide were detected. At pH 7.5, higher released peptide concentrations were measured in Tris-HCl buffer (25 mM) compared to phosphate buffers, and only traces were detected in PBS. As concluded for Oxm, these results show that electrostatic interactions play a major role in the stability of fibrillar Aib2-Oxm. Interestingly, Aib2-Oxm fibrils have a higher propensity to dissociate than Oxm fibrils and require higher salt concentrations to remain stable in solution. As the net charge of Oxm and Aib2-Oxm peptides is similar and not affected by the Aib2 mutation, this result can be explained by a different conformational state of the Aib2-Oxm peptide within the fibrils, as shown in the structural studies, due to the presence of the Aib residue which is known to be a strong helix inducer in peptides.

**Figure 5:**
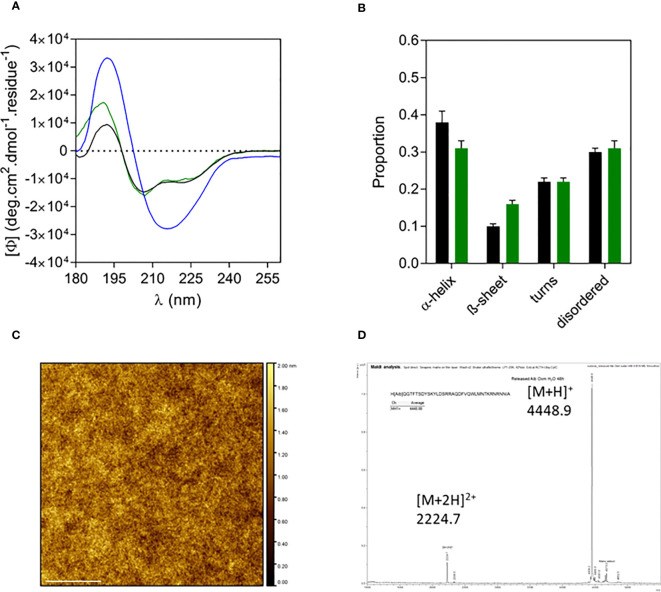
Characterization of released Aib2-Oxm. a-d, Far-UV CD spectrum **(A)** and analysis **(B)** of free (black) and fibrillar Aib2-Oxm (blue) in 0.09% saline and released Aib2-Oxm (green) in water, examples of a representative AFM image **(C)** mass spectrum **(D)** of released Aib2-Oxm in water. Scale bar, 1 μm.

Similar to Oxm, the far-UV CD spectrum and analysis of released Aib2-Oxm in water showed that the peptide recovered its initial conformation, which is characterized by helical and disordered structures in proportions similar to free peptides (**Figures 5A, B**). The AFM images of released peptide in water showed a smooth layer of peptide spread onto mica (**Figure 5C**). As measured by mass spectrometry, the peptide remained chemically intact after release from the nanofibrils ([M+H]^+^ = 4448.88, **Figure 5D**).

**Figure 6:**
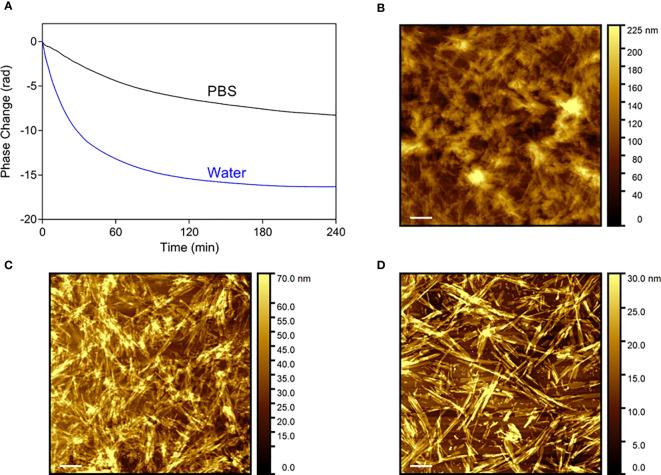
Dissociation profile of Aib2-Oxm nanofibrils. **(A)** Phase changes as a function of time on top of the DPI sensor coated with collagen and fibrillar Aib2-Oxm in water (blue) and PBS (black). b-d, Representative AFM images of Aib2-Oxm nanofibrils deposited onto a collagen layer before **(B)** and after incubation in PBS **(C)** and water **(D)** for 18 h. Scale bar, 1 μm.

A surface-based technique, dual polarisation interferometry (DPI), was used to assess the dissociation profile of Aib2-Oxm fibrils under similar conditions that the ones used with Oxm, i.e. conditions mimicking physiological pH, ionic strength and temperature, with the fibrils in interaction with one main component of the s.c. space (collagen), and with peptide cleared upon release from the fibrils (9). A decrease in phase change as a function of time is observed under these conditions, indicating the removal of material from the surface. As observed for Oxm, a sharper decrease was monitored during the first 4 h in water than in PBS pH 7.4 (**Figure 6A**). However, a significant decrease was also monitored in PBS and quantification of the change in mass after 4 h incubation showed that 38 ± 7% of the deposited material was removed from the surface in water and 19 ± 2% was removed in PBS when only 10 ± 2% was observed for Oxm in similar conditions (9). This shows that in PBS, Aib2-Oxm fibrils have a higher propensity to dissociate than Oxm fibrils. AFM images of the surface before (**Figure 6B**) treatment, and after flowing with PBS (**Figure 6C**) or water (**Figure 6D**), show that the removal of materials corresponded to a dissociation of the fibrils.

### The released Aib2-Oxm peptide is active, GLP-1R biased and non-toxic *in vitro*

**Figure 7:**
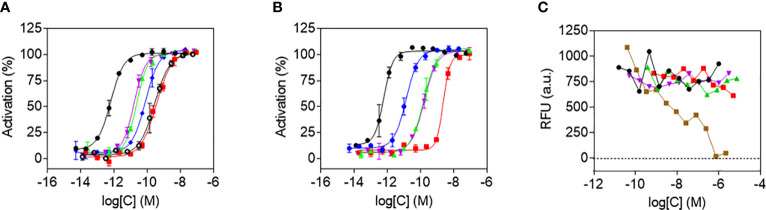
Agonist potency and cytotoxicity profiles of Aib2-Oxm species. a and b, *In vitro* potencies determined in cAMP accumulation assays in CHO cell lines expressing human GLP-1 **(A)** and GCG **(B)** receptors of free Aib2-Oxm (green), released Aib2-Oxm (violet), fibrillar Aib2-Oxm (red), free Oxm (blue), glucagon (black), GLP-1 (black, open circles). Data show representative curves of > 5 independent experiments. Curve data are the arithmetic mean ± s.d. of duplicate data points. **(C)** Forty-eight-hour cytotoxicity prolife in CHO-GLP-1R cells of vehicle (black), free Aib2-Oxm (green), released Aib2-Oxm (violet), fibrillar Aib2-Oxm (red), Staurosporine (brown). RFU, Relative fluorescence units. Data show representative curves from 3 independent experiments.

The functional potency of released Aib2-Oxm was determined in comparison to that of free and fibrillar Aib2-Oxm using cAMP accumulation assays in Chinese hamster ovary (CHO) cells expressing recombinant human GLP-1 (hGLP-1R) or glucagon (hGCGR) receptors (**Figures 7A, B**). Free and released Aib2-Oxm were full agonists in hGLP-1R- and hGCGR-expressing cells, compared to the maximum effects of GLP-1 or glucagon peptides in GLP-1R and GCGR assays, respectively. We have discussed previously that formation of fibrillar structures should preclude pharmacological activity of the peptide, therefore *in vitro* activity of the fibrillar material results from *de novo* release of peptide in the assay conditions (9).

Released and free Aib2-Oxm had comparable potencies against hGLP-1R, with EC_50_ values of 10.2 ± 1.9 (geometric mean ± s.e.m.) and 9.4 ± 3.1 pM, respectively, whereas fibrillar Aib2-Oxm was approximately 20-fold less potent with an EC_50_ of 176.2 ± 78.3 pM (**Table 1**). In the hGCGR-expressing line released and free Aib2-Oxm also showed similar potencies, with EC_50_ values of 183.9 ± 41.3 and 154.7 ± 34.2 pM respectively and fibrillar Aib2-Oxm was again greater than 10-fold less potent, EC_50_ 2367.7 ± 296.2 pM (**Table 1**). Additionally, when released Aib2-Oxm potency was compared to released Oxm potency (EC_50_ values of 54.8 ± 14.6 and 14.7 ± 3.6 pM at h GLP1-R and h GCGR respectively, **Table 1**) it was found to be more potent than Oxm at GLP1R but less potent on GCGR. This switch in glucagon/GLP1 relative potency balance from 2.6 to 0.06 for Oxm and Aib2-Oxm respectively makes Aib2-Oxm a GLP-1 biased dual agonist similar to published dual agonists being pursued as type 2 diabetes therapeutics (14, 15).

**Table 1:** Summary of peptide potency in cAMP accumulation assay at hGLP-1R and hGCGR expressed in CHO cell.

	hGLP-1R EC50 (pM)		hGCGR EC50 (pM)	
	Geomean	s.e.m.	Geomean	s.e.m.
**GLP-1 (7-36) amide**	0.8	0.1	NA	–
**Glucagon**	142.5	44.3	0.6	0.1
**Oxm**	54.8	14.6	14.7	3.6
**Free Aib2-Oxm**	9.4	3.1	154.7	34.2
**Fibrillar Aib2-Oxm**	10.2	1.9	183.9	41.3
**Released Aib2-Oxm**	176.2	78.3	2366.7	296.2

Data is geometric mean (Geomean) and standard error of mean (s.e.m.) from 3-4 independent experiments. NA, not active.

To assess the toxicity of Aib2-Oxm species, a cell viability assay was performed in the presence of free, released and fibrillar Aib2-Oxm. Metabolic bioactivity of living cells was measured through bioreduction of a resazurin-based dye (**Figure 7C**). None of the forms of Aib2-Oxm were cytotoxic at concentrations up to 1000-fold above the EC_50_ value in the CHO-hGLP-1R cell line. The positive control cytotoxic agent staurosporine, a protein kinase C inhibitor, showed the expected effect on loss of cell viability. These studies show that released and fibrillar Aib2-Oxm are not acutely cytotoxic at the concentrations tested and the fibrils can be used as a reservoir in which active and nontoxic peptides can be stored and released upon dissociation of the fibrils.

### Fibrillar Aib2-Oxm prolongs exposure in serum following s.c. administration

**Figure 8:**
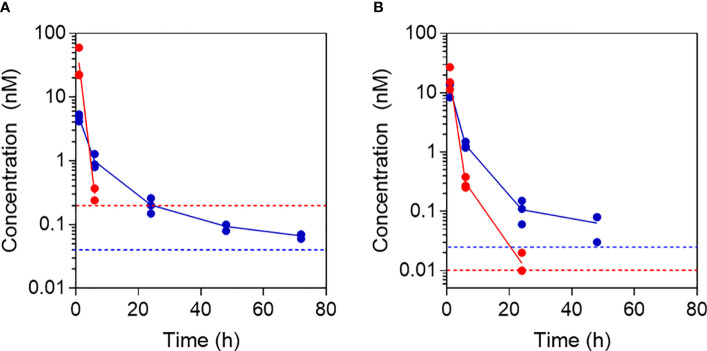
Pharmacokinetic profiles following administration of free and fibrillar Aib2-Oxm. Aib2-Oxm bioactivity in rat **(A)** or mouse **(B)** serum determined using *in vitro* cell-based cAMP bioassay for determining GLP-1 receptor agonist bioactivity after s.c. administration of 10 mg kg-1 of free Aib2-Oxm (red) or fibrillar Aib2-Oxm (blue). n=3 animals dosed with each material. Data shown as mean (line) and individual points above LOQ (dotted lines).

To investigate the ability of fibrillar Aib2-Oxm to release bioactive peptide *in vivo*, we administered either the fibrillar or free Aib2-Oxm formulations at 10 mg kg^-1^ s.c. in both mouse and rats and took serum samples for up to 5 days post-injection. Serum Aib2-Oxm content was determined as *in vitro* bioactivity using cell-based hGLP-1R cAMP bioassays (**Figure 8**). In rats, 1 h after s.c. dosing with free Aib2-Oxm (**Figure 8A**), approx. 31nM serum exposure was achieved, which is 30-fold higher than that seen after the equivalent dose of Oxm in our previous report (9), and is consistent with the reported protection from protease degradation. Administration of fibrillar Aib2-Oxm 10 mg kg^-1^ s.c. in the rats resulted in Aib2-Oxm bioactivity out to 3 days, the final time point we analysed, at 0.05 nM whereas after administration of free Aib2-Oxm, no cAMP assay activity could be quantified past the 24 h timepoint. Overall, this shows a sustained release of Aib2-Oxm from the fibrils *in vivo* that is characterised by a significantly longer bioactivity than free Aib2-Oxm. A similar study conducted in mice (**Figure 8B**), the species used for subsequent pharmacodynamic studies, showed the same profile, with extended serum bioactivity in the cAMP assay after administration of the fibrillated formulation compared to the solution formulation.

### s.c. administration of Aib2-Oxm fibrils reduced food intake up to 72h post-injection

**Figure 9:**
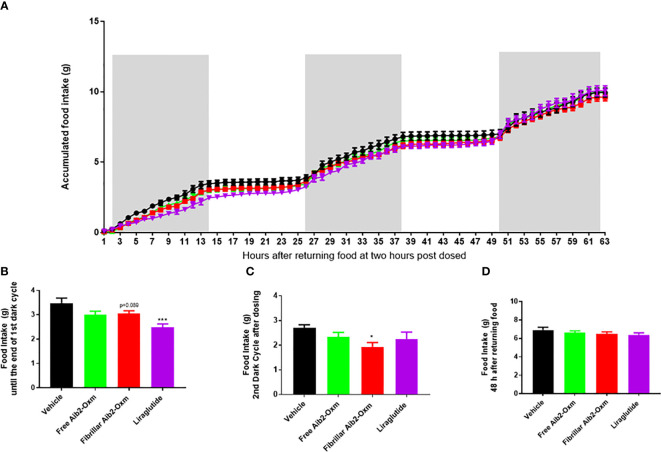
Effect of fibrillar Aib2-Oxm on food intake. a-d, Effect of fibrillar Aib2-Oxm (red, 15 mg.kg-1) vs vehicle (black), free form Aib2-Oxm (green, 15 mg.kg-1) and liraglutide (purple, 40 µg.kg-1) at single injection site on food intake in C57B6J lean mice. Accumulated food intake in lean mice **(A)**, food intake from returning the food until end of the dark cycle [first 14-hour, **(B)**] and 2nd dark cycle **(C)**, accumulate 48h food intake **(D)**. Statistic was carried out with One-way ANOVA by Dunnett’s. * and *** are P ≤ 0.05 and 0.001 when compared to vehicle control respectively. p=0.089 vs vehicle by unpair T test. n=8 per group.

The propensity of fibrillar Aib2-Oxm to produce a pharmacological effect *in vivo* was then assessed by monitoring food intake in mice, which is one of the most significant pharmacodynamic effects reported for this class of peptides. Liraglutide, used as a positive control at 40 µg kg^-1^, significantly reduced food intake as expected in the first 12 hours (5pm to following morning 5am, **Figures 9A, B**) after returning the food but such an effect was lost at the second dark cycle (7pm to following morning 7am, **Figures 9A, C**). When mice were dosed with 15 mg kg^-1^ of fibrillar Aib2-Oxm, food intake was significantly reduced during the first 12 hours after returning to food (**Figures 9A, B**) as well as at the second dark cycle (**Figures 9A, C**) but such an inhibition was not observed as accumulated 48 h food intake (**Figures 9A, D**). This inhibition pattern can be explained by the prolonged presence of pharmacologically active Aib2-Oxm peptide after being released from the fibrils.

**Figure 10:**
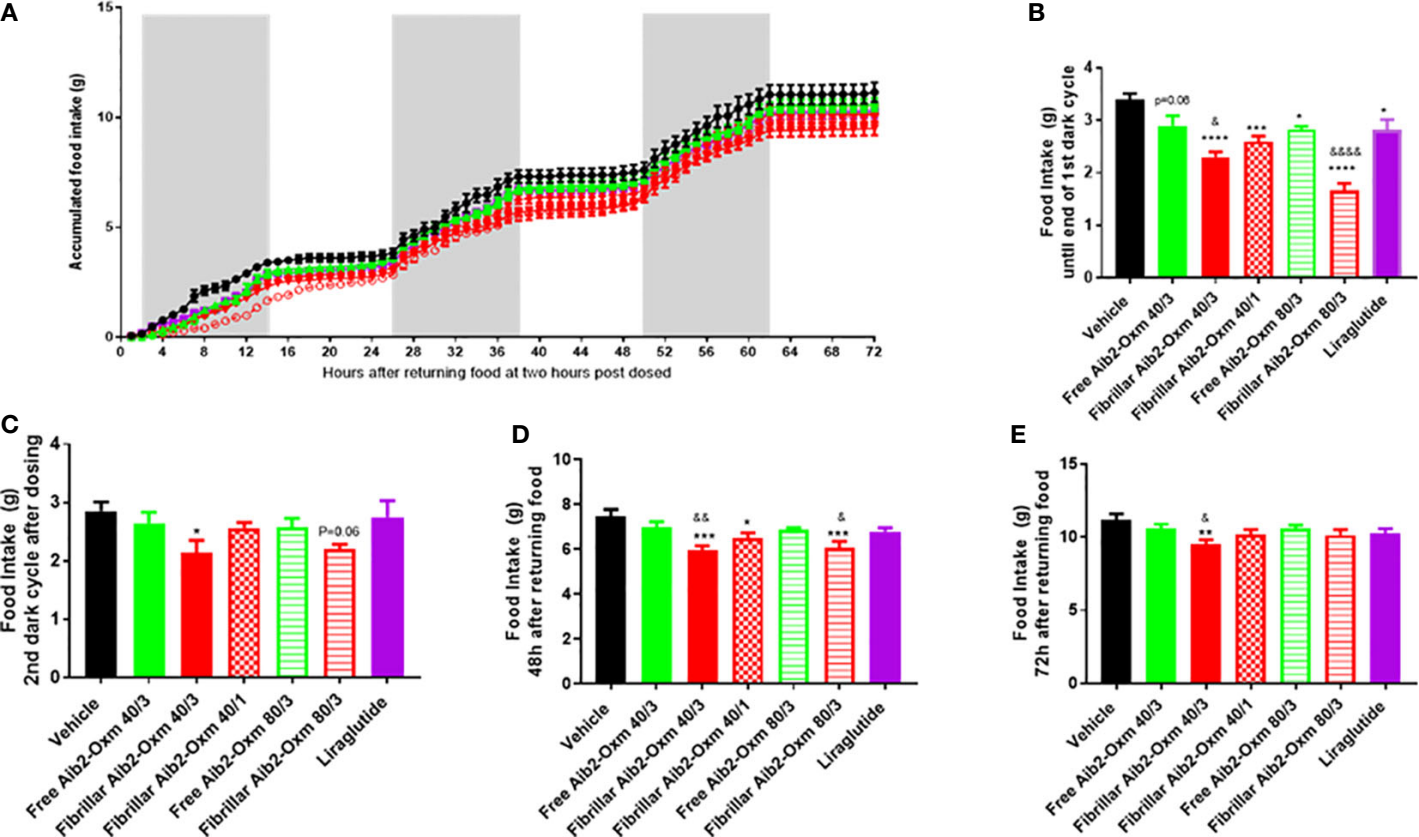
Effect of fibrillar Aib2-Oxm vs free form Aib2-Oxm at 40 or 80 mg.kg-1 at single or multiple injection sites on food intake in C57B6J lean mice. **(A)**, Mean accumulated food intake in lean mice. From returning the food until end of the first dark cycle (First 14-hour, **B**) and second dark cycles **(C)** of food intake after returning food. Food intake at 48 hours **(D)** and 72 hours **(E)** after returning food. Statistics was carried out with One-way ANOVA by Dunnett's. *, **, *** and **** are P ≤ 0.05, 0.01, 0.001 and 0.0001 when compared to vehicle control respectively. &, &&, and &&&& are P ≤ 0.05, 0.01, 0.001 and 0.0001 when compared to the same doses of free Aib2-Oxm respectively by unpaired t test. Liraglutide was dosed at 40 µg.kg-1; Free Aib2-Oxm 40/3: free Aib2-Oxm at 40 mg.kg-1 over 3 sites. Fibrillar Aib2-Oxm 40/3: fibrillar Aib2-Oxm at 40 mg.kg-1 over 3 sites. Fibrillar Aib2-Oxm 40/1: fibrillar Aib2-Oxm at 40 mg.kg-1 over 1 site. Free Aib2-Oxm 80/3: free Aib2-Oxm at 80 mg.kg-1 over 3 sites. Fibrillar Aib2-Oxm 80/3: fibrillar Aib2-Oxm at 80 mg.kg-1 over 3 sites. n=8 per group.

In a second set of pharmacodynamic experiments, the doses were increased by a combination of higher injection volumes, greater peptide concentrations and increasing the number of injection sites. As expected, all the dosing groups showed significant reduction in food intake during the first 12 hours after returning food (**Figures 10A, B**) and such an inhibition was lost in liraglutide group during the second dark cycle (**Figures 10A, C**). Interestingly, fibrillar Aib2-Oxm at 40 mg kg^-1^ dosed at multiple sites still significantly inhibited food intake during the second dark cycle (**Figures 10A, C**), which was lasted until 48 hours (**Figures 10A, D**) and 72 hours post dose (**Figures 10A, E**). However, such a magnitude of effect was not seen for free Aib2-Oxm at the same dose (**Figures 10A–D**). Moreover, 80 mg.kg^-1^ of fibrillar Aib2-Oxm achieved with multiple injection site administration also demonstrated significant inhibition on food intake during the first dark cycle (**Figures 10A, B**) and up to 72 hours (**Figures 1A, D**) post-dose. Such an effect was not observed for the free Aib2-Oxm single dose nor for same dose of free Aib2-Oxm dosed at multiple injection sites (**Figures 10A, C**).

## Discussion

Gut hormones and dual agonist peptides, such as Oxm, have a number of beneficial effects on nutrition and metabolism including increased energy expenditure, reduced body weight gain, enhanced insulin secretion and improved glucose homoeostasis (3). Despite its many advantages as a potential therapeutic agent, Oxm is subjected to rapid renal clearance and protease degradation limiting its clinical application. Previously, we have investigated the self-assembly propensity of Oxm and explored the application of fibrillar Oxm as a long-lasting formulation (9). We have shown that s.c. administration of fibrillar Oxm can significantly prolong its bioactivity *in vivo* from a few hours to a few days. In addition, s.c. administration of fibrillar Oxm in mice produced a pharmacological effect on glucose lowering. However, despite the benefit of using fibrillar Oxm propensity to release bioactive peptide and prolong peptide presence in serum, Oxm is still subject to protease degradation upon release from the fibrils. In the process of optimizing this approach, we have applied this strategy to an analogue of Oxm, Aib2-Oxm, for which the Ser residue in position 2 is replaced by an Aib residue. Aib2-Oxm peptide has been shown to be resistant to DPP-4 degradation and induced a significant appetite suppressive effects in mice (12). In addition, we were interested in assessing the effect of introducing a helix inducer in the peptide sequence on the reversible self-assembly propensity. As Oxm and Aib2-Oxm peptides have similar isoelectric points, the difference in the self-assembly propensity and stability would originate from a difference in the conformational state and/or packing of the peptide within the fibrils. The biophysical characterization and QCM-D experiments show that in comparison to Oxm: (1) Aib2-Oxm fibril elongation process requires higher ionic strength solutions; (2) Aib2-Oxm elongation rate is significantly slower; (3) Aib2-Oxm has a different conformational state within the fibrils; (4) Aib2-Oxm fibrils have different morphologies with twisted and ribbon like structures and (5) Aib2-Oxm fibrils have a higher propensity to dissociate. Therefore, even if the Aib residue did not prevent the self-assembly process, it appears that the equilibrium between free peptide and fibrils is more shifted toward free peptide for Aib2-Oxm as compared to Oxm.

The Aib-Oxm residue has a clear impact on the *in vivo* PK properties of the peptide as can be seen from the exposure (calculated using a separate standard curve for each peptide) following equivalent doses of free Oxm and free Aib2-Oxm in the rat, with ~30-fold greater bioactivity after1 h after dosing with Aib2-Oxm versus Oxm. After s.c. dosing with fibrillar Aib2-Oxm in the rat, there is a 5-fold increase in serum bioactivity relative to our previous study with fibrillar Oxm. This result could be explained by the higher chemical stability of Aib2-Oxm peptide in serum, particularly the reported resistance to DPP-4 mediated degradation13. While we do not have a clear mechanistic explanation for this observation, we observed differences in the way the fibrillar forms of these two peptides behave *in vivo*, with a clear advantage to Aib2-Oxm. The higher exposure in serum is reflected in the significant PD effect that was observed on food intake when the fibrils are administered subcutaneously, with a long-lasting effect observed up to 72 hours post-injection. This study shows that by combining reversible self-assembly propensity with peptide protease resistance property, these peptides can achieve a long-lasting PD effect *in vivo*. This study demonstrates a very promising approach to develop self-assembly long acting formulations for this class of peptides which are highly investigated to treat obesity and T2DM. Further optimization of the peptide sequence should enable a significant reduction in dosing frequency by optimising fibril release rate and peptide potency.

## Methods

### Materials

Synthetic (Aib2) - oxyntomodulin (His-Aib-Gln-Gly-Thr-Phe-Thr-Ser-Asp-Tyr-Ser-Lys-Tyr-Leu-Asp-Ser-Arg-Arg-Ala-Gln-Asp-Phe-Val-Gln-Trp-Leu-Met-Asn-Thr-Lys-Arg-Asn-Arg-Asn-Asn-Ile-Ala, acetate salt, MW = 4447.9 g.mol^-1^) (≥ 95% purity) and Synthetic native human oxyntomodulin (His-Ser-Gln-Gly-Thr-Phe-Thr-Ser-Asp-Tyr-Ser-Lys-Tyr-Leu-Asp-Ser-Arg-Arg-Ala-Gln-Asp-Phe-Val-Gln-Trp-Leu-Met-Asn-Thr-Lys-Arg-Asn-Arg-Asn-Asn-Ile-Ala, acetate salt, MW = 4449.9 g mol^-1^) (≥ 95% purity) were purchased from Bachem (Switzerland). HPLC grade water (resistivity >18 MΩ cm), PBS (pH 7.4), phosphate, Tris-HCl, sodium dodecyl sulfate (SDS) and human collagen (Bornstein and Traub type I, recombinant, expressed in *Nicotiana*) were purchased from Sigma–Aldrich (UK). Sterile saline (0.9% (*w v^-1^*) sodium chloride solution) (Baxter) and sterile water for injection BP (Fresenius Kabi) were purchased from VWR (UK). Millex syringe filters (pore size = 0.22 µm), Ultrafree-MC centrifugal filter devices with Durapore membranes (pore size = 0.22 µm) and Millipore Amicon Ultra-0.5 50K centrifugal filter devices were obtained from Fisher Scientific (UK). Hellmanex III was obtained from Hellma Analytics (Germany). Clear glass vials were obtained from MedImmune Nijmegen manufacturing facility (Netherlands).

### Preparation of fibrillar Aib2-oxyntomodulin

The preparation of Aib2-Oxm solutions was carried out in a class 2 cabinet using sterile glass vials. Aib2-Oxm was dissolved at 10-20 mg mL^-1^ in sterile NaOH (4.3 mM) and 0.09% saline solution (diluted from the 0.9% saline solution) to achieve a pH between 7.0 and 7.3. The solution was passed through a 0.22 µm pore size membrane, and peptide concentration was measured before diluting the solution to 10 mg mL^-1^ using 0.09% saline. The solution was then incubated at 37°C for 3 to 6 days with orbital shaking (200 rpm, ThermoScientific MaxQ 4450 benchtop orbital shaker) and then 2–3 days without agitation. Thereafter, 1 mg mL^-1^ of fibrillar Aib2-Oxm was incubated with 10 mg mL^-1^ free peptide in 0.09% for 1 week at RT without agitation. Finally, the 10 mg mL^-1^ fibrillar Oxm solution was diluted to varying concentration between 1 to 4 mg mL^-1^ using sterile 0.09% saline, incubated at RT for 2–9 days before being stored at 4°C. The conversion yield was assessed by measuring the concentration of the remaining free peptide. The fibrillar material was separated from the free peptide in solution after centrifugation of an aliquot of the solution for 30 min at 16 200 x g and filtering the supernatant through a 50 kDa molecular weight cut-off membrane.

### QCM-D studies

QCM-D experiments were performed on a QSense E4 Analyzer (Biolin Scientific) equipped with a High Precision Multichannel Dispenser (Ismatec). We used silicone dioxide (SiO2) coated QSX303 chip sensors (Biolin Scientific). Sensor preparation. Prior to experiments, sensors were cleaned by immersion in 2% (w/v) SDS and incubation at 37°C for 45 min. Sensors were rinsed with Ethanol and water, dried in a stream of Nitrogen, and cleaned in a UV/ozone ProCleanerTM (Bioforce Nanosciences) for 20 min. Sensors were either used without modification (blank), or with short fibrils (seeds) physically adsorbed to the surface by drying of a seed dispersion on the SiO_2_ layer. In order to confine deposited sample drops to the active surface of the chip during drying, a custom-made PTFE block with a circular hole matching the active surface was clamped to each chip. For creating seed samples with matching fibril lengths, aliquots of fibril stocks were fragmented by sonication in a FB15049 bath sonicator (Fisher Scientific). The sonication time was adjusted to the length of fibrils in stock samples to deposit approximately equal numbers of Oxm - and Aib2-Oxm fibrils. Seed dispersions were diluted to 0.1 mg mL^-1^ in HPLC grade water (Fisher), and 40 μL dilution was pipetted on each sensor. Sensors were dried overnight and imaged by AFM (see below) before installation in the QCM-D machine. Sensors were equilibrated at 25°C in 0.09% saline for at least 1 h before buffer or freshly prepared peptide solutions were flown over the surface at a rate of 0.1 mL min^-1^. Resonance frequency shifts were recorded at >83 measurements per minute. Only the third resonance frequency overtone f_3_ was used for analysis. The first two minutes of interaction are dominated by adsorption of soluble peptide to the surface of the sensor, and not included in the analysis shown here. Linear frequency decrease after physisorption indicates mass deposition by elongation of fibrils. We used the slope of frequency change during the 10 min following adsorption as a measure of elongation kinetics. To exclude frequency shifts by multilayer deposition or aggregation during the experiments, we exposed a fibril-free (blank) SiO_2_ chip to a solution of 0.5 mg mL^-1^ Oxm. We performed triplicate experiments where independently fragmented Oxm or Aib2-Oxm fibrils were exposed to 2 mg mL^-1^ Oxm or Aib2-Oxm, respectively. For comparison purpose, the Oxm experiment was additionally performed at 0.5 mg mL^-1^. We further flowed 0.5 mg mL^-1^ Oxm over Aib2-Oxm fibrils in order to investigate cross-seeding. As a control for the integrity of fibrils during the experiment, a sensor with Aib2-Oxm fibrils was flushed with 0.09% saline in parallel to one of the experiments.

### AFM study

A PicoPlus AFM instrument with a PicoSPM II controller from Molecular Imaging (Agilent) was used for the AFM imaging studies. Images were acquired at room temperature in air using the AC mode with NSC36/no Al cantilevers (Mikromasch, with force constants varying from 0.6 to 2 N m^-1^). For each imaging experiment, an aliquot of the corresponding solutions was deposited onto freshly cleaved mica and left to dry without rinsing.

### Spectroscopic analysis

Peptide concentrations were measured using a NanoDrop 2000 UV/Vis spectrophotometer (Thermo Scientific, UK). Far-UV circular dichroism spectra (wavelength range: 260 to 180 nm) were acquired at 22°C on a Jasco J-815 spectropolarimeter using a 0.1 mm path length cuvette, with a data pitch of 0.5 nm, a 1-nm bandwidth, a scanning speed of 50 nm min^-1^, a 4-s response time, and a 5-scan accumulation. Buffer spectra were measured under the same conditions and subtracted from the sample spectra. Circular dichroism spectra were deconvoluted with the CONTINLL, SELCON3 and CDSSTR algorithms using CDPro software (16, 17). ATR FT-IR spectra of free and fibrillar Oxm were recorded in a Perkin Elmer Spectrum 400 FT-IR spectrophotometer equipped with a Specac’s Golden Gate™ ATR sampling accessory. A 2-μl sample of free and fibrillar Oxm (1 mg ml^-1^ in 0.09% saline) was placed on top of the ATR accessory and dried with a stream of nitrogen. This procedure was repeated 3 times. For each sample, 32 interferograms were co-added at 2 cm^–1^ resolution within the range of 1000 to 2000 cm^-1^. ThT and Trp fluorescence measurements were measured with a Hitachi Fluorescence Spectrophotometer F-700 Microplate. Trp fluorescence of peptide samples in the wells of the microplate was measured at 25°C with excitation at 277 nm and monitored at 285-500 nm. After Trp fluorescence measurement, ThT binding was monitored by adding ThT (50 µM) to the wells and exciting the sample at 440 nm and recording the emission fluorescence spectrum from 450 to 600 nm.

### Dissociation studies

Solutions of fibrillar Aib2-Oxm at 1 mg mL^-1^ in phosphate buffer (25 mM, pH 7.5 and pH 6), Tris-HCl buffer (25 mM, pH 7.5), water (pH 5.9 to 6.2), 0.09% and 0.18% saline (pH 5.9 to 6.2), PBS and aqueous HCl (10 mM, pH 2) were incubated for 4 h and 48 h under quiescent conditions at 37°C. The samples were first centrifuged at 16 200 x g for 30 min. The collected supernatant was then filtered through a 50 kDa molecular weight cut-off membrane. The concentration of peptide was measured in the filtrate and compared to the initial peptide concentration to assess the percentage release. Released peptides in water were used for further studies. Mass spectra were obtained by matrix-assisted laser desorption ionisation (MALDI) on a Bruker ultrafleXtreme mass spectrometer. The net charge of the peptide versus pH was calculated using GPMAW 9.52a software.

### Dissociation study of fibrillar Oxm using DPI

A dual polarisation interferometer (Farfield Analight 4D, Biolin Scientific AB) was used to optically characterize the dissociation profile of fibrillar Oxm deposited onto collagen-coated sensor chips. Details of the instrumentation has been described previously (18). Human collagen, diluted to 0.2 mg mL^-1^ in water, was first deposited onto the unmodified oxynitride sensor chip and left to dry at room temperature before being briefly rinsed with water. Then, fibrillar Aib2-Oxm (1 mg mL^-1^, 10 µL) in 0.09% saline was deposited onto the collagen layer and left to dry at room temperature without rinsing. Water or PBS solutions were continuously flowed over the deposited fibrillar Oxm layer at a rate of 20 µL min^-1^ at 37°C. After incubation, aqueous 10 mM HCl (pH 2) and 2% Hellmanex in water were injected to remove any residual materials (including the collagen layer) before proceeding to the chip calibration using 80% (*w w^-1^*) ethanol in water. Data were analysed using the Analight Explorer 1.6.0.27583 (Farfield-Biolin Scientific AB, Sweden) to calculate the layer refractive index, density, thickness and mass (18). Variations in the layer properties were calculated from the maximum values at the start of incubation.

### Potency assay

CHO cells stably transfected with human GLP1R or human GCGR receptor were used to determine *in vitro* agonist potencies of free, released and fibrillated Oxm or Aib2-Oxm in cAMP accumulation assays as previously described (Ouberai et al., 2017). In brief, cells were plated at 500 cells per well in 384-well black shallow microtiter plates (Corning, USA) and incubated with serially diluted peptide samples for 30 min prior to lysis and detection using the HTRF cAMP dynamic 2 assay kit as per manufacturer’s instructions (Cisbio, France). Plates were read on an Envision plate reader (Perkin Elmer, USA). Eleven-point concentration-response curves were generated in duplicate for at least four independent experiments, and data were represented as the percent activation of the maximum reference ligand. Curves were fitted using nonlinear regression analysis in GraphPad Prism software 6.03 (GraphPad, USA).

### Cell viability assay

CHO-hGLP-1R cells described above were plated at 10 000 cells per well in 96-well black clear-bottom poly-d-lysine-coated microtiter plates (Corning, USA). Growth medium comprised DMEM supplemented with 10% FBS (Sigma-Aldrich). Cells were pre-treated with free, released and fibrillated Aib2-Oxm or cytotoxic standard staurosporine (Sigma-Aldrich) for 48 h prior to incubation for 5 h with resazurin dye (*in vitro* toxicological assay kit, Sigma–Aldrich) at 37°C in a 5% CO_2_ atmosphere. Fluorescence emission was measured at 590 nm (560 nm excitation) using an Envision plate reader (Perkin Elmer, USA).

### Pharmacokinetic studies

All animal care and experimental procedures were performed in accordance with the Animal (Scientific Procedures) Act 1986, local establishment usage guidelines, and ARRIVE guidelines. The project Licences authorising the work were approved by a local ethical review body (AWERB). Animals were sourced from Charles River UK, and group housed in individually ventilated cages within a barrier unit with 12-hour light/dark cycle (7am-7pm) and ad libitum access to chow diet and water. The study designs included n=3 samples per PK timepoint so that we could obtain a clear estimate of the pharmacokinetic profile. For mouse PK experiments, a total of 18 male C57BL6/J mice age at 6-8 weeks were randomly divided into two groups and dosed with 10 mg.kg^-1^ of fibrillar or free form of Aib2-Oxm by s.c. at 5 ml.kg^-1^ with 2 mg.ml^-1^ dosing solutions. Blood for serum was collected *via* a tail vein from three mice from either group at 1, 6 and 24 hours, and terminal serum from all mice at 48 hours post dose. Serum samples were stored in -80 degrees C. For rat PK experiments, 6 male CD rats weight at around 275 g were used and were randomly divided into two groups and were dosed with 10 mg.kg^-1^ of fibrillar or free form of Aib2-Oxm by s.c. at 5 ml.kg^-1^ with 2 mg.ml^-1^ dosing solutions. Blood for serum was collected *via* a tail to vein at time points of 1, 6, 24, 48 and 72 hours post dose. Serum samples were stored in -80 degrees C. Serum peptide content was determined as apparent concentration using an *in vitro* cell-based cAMP bioassay (Cisbio, France) to estimate ex-vivo agonist bioactivity in serum samples at human GLP1R as previously described (9). In brief, CHO K1 cells stably transfected with human GLP1R were used to compare the degree of cAMP accumulation in serum samples from treated animals against a standard curve generated by spiking peptide into naïve serum. Reference curve data were analyzed using nonlinear regression analysis, from which test serum samples were interpolated to give apparent concentration using GraphPad Prism.

### Pharmacodynamic studies

All animal care and experimental procedures were performed in accordance with the Animal (Scientific Procedures) Act 1986, local establishment usage guidelines, and ARRIVE guidelines. The project licences authorising the work were approved by a local ethical review body (AWERB). Male C57/Bl6 mice were sourced from Charles River UK, and group housed in individually ventilated cages within a barrier unit with 12-hour light/dark cycle (7am-7pm) and ad libitum access to chow diet and water. For experiments, 32 or 56 C57BL6/J mice (n=8/group) for the first and second study respectively, weight between 20-25 g, were single housed in the BioDaq cages for acclimatisation for 5 days before first pre-dosing subcutaneously (s.c) with total amount 100μl of saline at a single site or three sites respectively. Animals were randomised into treatment groups according to body weight and two days of individual daily food intake data. On the experimental day, food was withdrawn from all mice for 4 hours before treatment was given by s.c. injections at one or three injection sites. 40µg.kg^-1^ of liraglutide was used for both studies as a positive control. Food was then returned one-hour post dose, and the food intake data collection recording commenced. The data was continuously recorded for 72 hours before termination. Data was analysed by Data Viewer software supplied by BioDaq. Dosing information: In the first study, 15 mg.kg^-1^ of fibrillar or free form of Aib2-Oxm were dose at 7.5 ml.kg^-1^ with dosing solution at 2 mg.ml^-1^. In the second study, mice were dosed: 40 mg.kg^-1^ using 2 mg.ml^-1^ dosing solution at one injection site giving a total dose of 20 ml.kg^-1^, or 40 mg.kg^-1^using 2 mg.ml^-1^ dosing solution injected across three sites at 6.67 ml.kg^-1^ each or 80 mg.kg^-1^ using 4 mg.ml^-1^ dosing solution injected across three sites at 6.67 ml/kg per site.

## Data availability statement

The raw data supporting the conclusions of this article will be made available by the authors, without undue reservation.

## Ethics statement

The animal study was reviewed and approved by Animal Welfare and Ethical Review Body.

## Author contributions

MO, AG, and MW conceived the project. MO, AG, DH, DB, DC conceived and designed the experiments. MO, SK, DH, LL, JN and performed the experiments. MO, AG, DH, DB, JN, DC analyzed the data. MO, AG, SK, DH, DB, DC, and MW wrote the paper. All authors contributed to the article and approved the submitted version.
